# Assessing quality of critical care during an ongoing health emergency—a novel approach to evaluate quality of care at Lebanese public ICUs during COVID-19

**DOI:** 10.1093/intqhc/mzae028

**Published:** 2024-04-06

**Authors:** Karim AbouNader, Ghada Abou Mourad, Georges Chalouhi, Alissar Rady, Johan Von Schreeb, Märit Halmin

**Affiliations:** Department of Global Public Health, Karolinska Institutet, Solnavägen, Stockholm 171 77, Sweden; The World Health Organization, Bloc left 4th floor, Glass building, Museum Square, Beirut 5391, Lebanon; Middle East Academy for Learning Health Systems, Beirut, Lebanon; The World Health Organization, Bloc left 4th floor, Glass building, Museum Square, Beirut 5391, Lebanon; Department of Global Public Health, Karolinska Institutet, Solnavägen, Stockholm 171 77, Sweden; Department of Global Public Health, Karolinska Institutet, Solnavägen, Stockholm 171 77, Sweden

**Keywords:** COVID-19, quality of care, checklist, twinning, monitoring, intensive care

## Abstract

**Background:** Quality of care has been systematically monitored in hospitals in high-income countries to ensure adequate care. However, in low- and middle-income countries, quality indicators are not readily measured. The primary aim of this study was to assess to what extent it was feasible to monitor the quality of intensive care in an ongoing health emergency, and the secondary aim was to assess a quality of care intervention (twinning project) focused on Intensive Care Unit (ICU) quality of care in public hospitals in Lebanon.

**Methods:** We conducted a retrospective cohort study nested within an intervention implemented by the World Health Organization (WHO) together with partners. To assess the quality of care throughout the project, a monitoring system framed in the Donabedian model and included structure, process, and outcome indicators was developed and implemented. Data collection consisted of a checklist performed by external healthcare workers (HCWs) as well as collection of data from all admitted patients performed by each unit. The association between the number of activities within the interventional project and ICU mortality was evaluated.

**Results:** A total of 1679 patients were admitted to five COVID-19 ICUs during the study period. The project was conducted fully across four out of five hospitals. In these hospitals, a significant reduction in ICU mortality was found (OR: 0.83, *P* < 0.05, CI: 0.72–0.96).

**Conclusion:** We present a feasible way to assess quality of care in ICUs and how it can be used in assessing a quality improvement project during ongoing crises in resource-limited settings. By implementing a quality of care intervention in Lebanon’s public hospitals, we have shown that such initiatives might contribute to improvement of ICU care. The observed association between increased numbers of project activities and reduced ICU mortality underscores the potential of quality assurance interventions to improve outcomes for critically ill patients in resource-limited settings. Future research is needed to expand this model to be applicable in similar settings.

## Introduction

The COVID-19 pandemic has challenged the provision of health care, strained resources and health care staff, as well as pronounced already existing health inequities [1]. COVID-19-infected patients overwhelmed health systems and revealed limitations in managing a large influx of critically ill patients while maintaining sufficient quality of care [2]. Within the plans for universal health coverage (UHC) and Sustainable Development Goal 3 (SDG 3), quality of care has been highlighted as an essential component for provision of adequate care. Specifically, it has become a core component for mortality reduction and attainment of UHC [3]. In low- and middle-income countries (LMICs), low quality of care is reported to be responsible for 5 million excess deaths, of which 55% could be averted by improved quality of care [3].

The Donabedian model of quality of care builds on the assessment of health care services through structure, process, and outcome indicators [4]. The model assumes that components in the structure and process influence the outcome [5]. While the measurement of health outcome data requires substantial resources and established systems for data collection, the measurement of the structure and process is presumably easier and feasible and can be more efficient in identifying quality gaps in need of improvement [6]. In intensive care units (ICUs) of high-income countries (HICs), quality of care is increasingly monitored and used to address gaps and benchmarking between units; however, the variation among the indicators used and their definitions is high. In LMICs, such monitoring is scarce due to a lack of resources and a lack of established systems for data collection, making it difficult to assess and improve the quality of care [6]. In Lebanon, a country categorized as a lower middle-income country [7], monitoring of quality of care in public hospitals has not been systematic, while in private hospitals, quality is more systematically monitored. Since 2019, the country has faced multiple challenges, including an economic crisis, the COVID-19 pandemic, and the Beirut Harbor blast in August 2020 [8]. In Lebanon, most care has traditionally been provided by private hospitals, and before the compound crises (economic and COVID-19 crises), 90% of hospital beds were in the private sector (unpublished data from the World Health Organization Regional Office for the Eastern Mediterranean). The public hospital system was underfunded with a lack of staff, equipment, and medications [9]. With these crises, patients were increasingly forced to seek care in public hospitals [10]. With the onset of the COVID-19 pandemic and a rapid economic decline, the health care system’s ability to care for critically ill patients has been progressively worsening [11]. Due to a massive exodus of physicians and nurses, the quality of care in ICUs in public hospitals has been identified as a cause of major concern [8]. To mitigate the predicted deterioration, the World Health Organization (WHO) initiated support, starting in 2020, to public hospital COVID-19 ICUs with the provision of nurses and essential equipment. Additionally, a targeted intervention to improve quality of care focusing on capacity building was developed and implemented by WHO to strengthen knowledge and skill building for ICU staff. A clear focus was placed on ICUs, as they were a cost-heavy and resource-intensive service at the frontlines of the COVID-19 pandemic with a massive increase in the influx of critically ill patients [12].

To date, there are limited available studies that assess the outcomes of ICU quality assurance interventions during ongoing health crises in LMICs. The primary aim of this study was to assess to what extent it was feasible to monitor the quality of ICU care in an ongoing health emergency, and the secondary aim was to assess a quality of care intervention, called the twinning project focused on ICU quality of care in public hospitals in Lebanon.

## Methods

### Study design

We conducted a retrospective cohort study nested within an intervention implemented by the WHO Regional Country Office through the Middle East Academy for Learning Health Systems (MEDALS) in close coordination with the Ministry of Public Health (MOPH). Data from public hospitals enrolled in the project were used.

### Studied intervention

The twinning project, a targeted intervention for improvement of quality of care, was designed and quickly implemented during the onset of the rapidly evolving COVID-19 pandemic and focused on strengthening clinical processes. This intervention was built on the assumption that knowledge sharing between better resourced facilities (private university hospital ICUs) and less resourced public ICUs would lead to improved outcomes. The intervention paired private university hospitals, adhering to international quality standards and clinical guidelines, with public hospitals in both rural and urban settings. The included public hospitals varied in the size of their ICUs, and the number of ICU beds also varied with time during the pandemic according to fluctuating needs. Each private university hospital involved sent on a regular basis an ICU team consisting of ICU physicians, ICU nurses, and quality officers trained in COVID-19 case management to assigned public hospital COVID-19 ICUs across the country. The focus was to implement standardized clinical protocols that did not previously exist in public ICUs and thereby improve the clinical care delivered in public hospitals’ COVID-19 ICUs. The protocols were in line with international standards and guidelines, with a clear focus on criteria for prone positioning and intubation, and adapted to the local context of each hospital. Matching pairs were made by the WHO coordinating team based on geographical proximity and previous relationships between hospitals. The twinning project used a decentralized approach where each hospital pair was tasked to design their own support plan based on evolving identified priority needs. Overall specific targets for evidence-based clinical procedures (prone position, intubation protocols, infection and prevention control measures, etc.) were set. The intervention was based on activities that included bedside coaching, common clinical rounds, development of protocols, and rotations of personnel between the paired hospitals.

### Monitoring system: quality of care checklist and outcome indicators

To assess quality of care throughout the project, a monitoring system framed in the Donabedian model and including structure, process, and outcome indicators was developed and implemented.

#### Quality of care checklist

First, a quality of care monitoring checklist was created using a systematic approach to assess quality of care in public COVID-19 ICUs at baseline and repetitively during the intervention to capture any changes over time. The checklist included 33 key indicators that assessed structure (7 indicators) and processes (26 indicators) (Supplementary Appendix 1). These indicators were selected through expert opinion and captured staffing, equipment, and clinical management [13]. The indicators were assessed regularly at predefined time points throughout the project, and a percentage score was obtained at each visit based on how many of the indicators were fulfilled.

The achievement of all indicators was considered an acceptable standard of quality of care. The development of the checklist has been described in detail in a previous publication [13]. When 80% of the predefined assessments were performed, it was considered a successful implementation of the checklist. Scoring of the checklist was conducted by two ICU specialists external to the hospitals.

#### Patient data

Second, a Microsoft Excel 16.7 register was set up to collect routine data, including key demographic data and ICU mortality, of all patients admitted to the ICUs of public hospitals between 1 January 2021 and 30 November 2021. Data were collected by medical staff on site daily, were entered in the register, and kept on secured computers in all ICUs taking part in the project. The register was sent monthly to the project coordination team at the WHO Lebanon office. Random crosschecking of reported variables with medical records was performed by the research team for validation.

### Data collection and study variables

The twinning intervention started at the end of December 2020 and lasted until November 2021. Data were collected at five out of six paired public hospital ICUs. One hospital included in the project refused to share their outcome data and was therefore not included in this study. The included hospitals were in five different governorates across Lebanon and varied in the number of COVID-19 ICU beds (from 3 to 21 beds). The available ICU beds in each hospital changed during the study period depending on the surge of COVID-19 cases. The inclusion criteria were patients admitted to a COVID-19 ICU with a positive SARS-CoV-2 PCR test. All five public hospitals owned their data and agreed to participate in this study. All personally identifiable information was removed before data were shared. No specific exclusion criteria were applied other than the exclusion of patients missing essential data.

We performed a regression analysis assessing the association between ICU mortality and the number of twinning activities. A twinning activity was defined as a private hospital team visit to a public hospital ICU with its associated activity (training, lectures, bedside coaching, etc.). Preemptively, the twinning project estimated approximately 100–120 visits to be completed between each hospital pair (approximately three visits per week) during the intervention. For twinning to be considered achieved, a hospital pair had to complete at least 30 activities. The key demographic and structural variables collected in the register were age (nominal), age group (categorical: 1–20 years, 20–40 years, 41–60 years, 61–80 years, and 81–100 years), sex (binary: male/female), and intubation status (binary: yes/no). Additional covariates were trimester (categorical: one to four), hospital (categorical: one to five), and ICU bed occupancy. ICU bed occupancy was categorized as low, medium, or high depending on the percentage of available beds that were occupied on a given date. Low ICU occupancy was between 0% and 50%, medium from 50% to 80% and high over 80%. We also performed sensitivity analyses to assess the effect of the completed twinning intervention on ICU mortality. The same covariates described above were used in a multivariate logistic regression to generate adjusted estimates.

All data analyses were performed using STATA v17.0. Adjusted odds ratios (aORs) were reported with 95% confidence intervals (95% CIs). The multivariate logistic regression of the intervention was computed accounting for all the covariates that were chosen stepwise and included in the analysis. Changes in checklist scoring from baseline are reported as percentages.

### Patient and public involvement

Patients were not directly involved in the design of the research question. However, the aim of the intervention described was patient-oriented by focusing on the quality of care delivered. The results of the study were disseminated to the involved hospitals and could support them in improving patient outcomes.

### Ethical considerations

Ethical approval for this study was obtained from St Joseph University Ethics Review Board in Lebanon (USJ-2021-243). All hospitals in the project gave written informed consent for data collection and monitoring. Each hospital owned their data, which were anonymized and shared with the investigators. The anonymized data were thereafter merged, analysed, and stored at WHO Lebanon. Patient privacy and confidentiality were thereby maintained throughout the study period, and no medical information on individual patients was disclosed or revealed. Analysis and reporting were performed at the group level, hence avoiding the risk of harm or integrity for individual patients as well as the public hospitals included. No individual was exposed to any harm due to this study.

The authors of this research were involved in the design and implementation of the twinning study but have no other conflicts of interest to declare.

## Results

### Baseline characteristics

**Figure 1:**
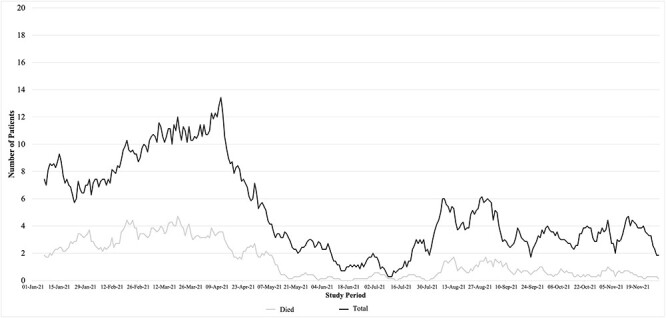
Distribution of admitted patients from 1 January 2021 to 30 November 2021 across the five public hospitals COVID-19 ICUs in the project.

From 1 January 2021 to 30 November 2021, a total of 1743 patients were admitted to the five COVID-19 ICUs included in this study (Fig. 1). Of the total sample, 64 patients were excluded due to missing data, leading to a final sample of 1679 patients. The characteristics of the study population are shown in Table 1. The age range was 4 to 100 years, and the median age [interquartile range (IQR)] was 63 years (38–88 years). Of the admitted patients, 54% were male. The median length of stay (IQR) was 5 days (3–7 days). The overall mortality was 30%. A total of 57 patients were transferred to other hospitals and thereby lost to follow-up but were counted as surviving to discharge in the mortality analysis.Change in checklist scoring.

**Table 1.** Demographic and clinical characteristics of the study population.

	Overall	Survivors	Non-survivors
Patients, n (%)	1679	1176 (70.0)	503 (30.0)
**Sex**			
Female, n (%)	773 (46.0)	530 (45.1)	243 (48.3)
Male, n (%)	906 (54.0)	646 (54.9)	260 (51.7)
**Age** (Median—Q1, Q3)	63 (38–88)	69 (50–88)	60 (34–86)
**ICU length of stay, days** (Median—Q1, Q3)	5 (3–7)	7 (3–12)	5 (3–7)

### Change in checklist scoring

The percentage of fulfilled indicators of the checklist before the start of the project defined the baseline of each hospital. Baseline scores ranged from 35% to 57% at baseline, with a mean score of 47% (SD 9.2). Throughout the study period and across all checklists, the mean improvement in the overall score was 20% (SD 0.36), with improvement in scores ranging from 17% to 25% from baseline to the end of the project (Table 2). Overall, across all hospitals, 80% of scheduled assessments were completed, and we observed that scores across all five hospital pairs increased from initiation to completion.

**Table 2.** Checklist score variation across hospital pairs.

Hospital pair	Number of checklist items completed	Initial score at baseline	Final score at completion	Score variation
Hospital Pair 1	5	34.6	73.9	34.6–76
Hospital Pair 2	3	57.0	72.0	57–72
Hospital Pair 3	4	44.0	58.0	44–58
Hospital Pair 4	4	68	70.8	68–81.8
Hospital Pair 5	4	52	68.0	34.8–68

### Twinning intervention and association with mortality

Each hospital pair conducted a varying number of activities based on availability. Hospital pair (HP) 1 conducted 54 activities, HP 2 conducted 13 activities, HP 3 conducted 61 activities, HP 4 conducted 30 activities, and HP 5 conducted 49 activities during the study period.

Among all patients, the odds of dying did not decrease with the twinning intervention during the study period (OR: 1.01, CI: 0.98–1.06, *P* > 0.05) (Table 3). A sensitivity analysis excluding HP 2, which completed only 13 twinning activities, not reaching our preset minimum target of interventions, revealed that the odds of dying decreased significantly with the twinning intervention (OR: 0.83, CI: 0.72–0.96, *P* < 0.05) among patients admitted to hospitals who had at least 30 activities performed during the twinning project (Table 4).

**Table 3.** Multivariate logistic regression analysis of the association of the twinning project with ICU mortality across all patients (*n* = 1679).

Twinning intervention	Crude OR (95% CI)	*P*	aOR^a^ (95% CI)	*P*
Across all patients (*n = *1679)	1.02 (0.98–1.06)	0.27	1.01 (0.93–1.10)	0.72

a Adjusted for covariates age, sex, ICU bed occupancy, and calendar time.

**Table 4.** Sensitivity analysis of twinning project association with ICU mortality across all patients of hospital pairs 1, 3, 4, and 5 (*n* = 1014).

Twinning intervention	Crude OR (95% CI)	*P*	aOR^a^ (95% CI)	*P*
Across patients of hospital pairs 1,3,4, and 5 (*n = *1014)	0.96 (0.92–1.01)	0.94	**0.83** (0.72–0.96)	**<0.01**

a Adjusted for covariates age, sex, ICU bed occupancy, and calendar time.

Notes: Bold text shows statistical significance.

## Discussion

### Statement of principal findings

Our study is the first attempt to monitor the quality of ICU care and to assess a quality of care intervention during a health emergency in a resource-limited setting. In a context where monitoring systems of patient outcomes were not established, our results demonstrated that despite the pandemic and the ongoing economic crisis, it was feasible to monitor and assess the quality of care. Additionally, this study indicated an association between the number of twinning activities and reduced ICU mortality. This suggests that an intervention that supported knowledge sharing and capacity building between private and public hospitals contributed to improvements in quality of care, despite a deteriorating situation in the country. However, it is hard to determine which component in this intervention had direct or indirect effects on quality of care.

### Strengths and limitations

When evaluating quality of care in health care settings, outcome measurements such as mortality are often measured as a proxy for overall patient health and well-being [14]. However, other measurements of quality of care, such as structure and process indicators, can be collected in a shorter timeframe and can provide additional information on the causes of poor outcomes [15]. Monitoring process and structure indicators (by our checklist) enabled rapid insight into the current quality of care and identified gaps that could be addressed. Through process measurements such as compliance with clinical guidelines, patient outcomes can be predicted [16]. This then provides another outlet towards improvement of patient outcomes through process indicators.

While quality of care in Lebanese hospitals has previously been assessed through hospital accreditation [17], patient satisfaction [18], and HCWs experience [19], this study is, to our knowledge, one of the first to assess quality of care using the Donabedian framework, including structure, process, and outcome.

Our study has limitations that must be considered. Our analyses lacked some important covariates that could not be accounted for. Mainly, nurse staffing, nurse-to-patient ratios, and vaccination rates were missing due to a lack of available data. Since the initiation of vaccine roll-out in Lebanon in February 2021, the variation in coverage across the population has been significantly influenced by vaccine hesitancy, leading to large disparities in vaccination rates across the country [20, 21]. A notable limitation to our study is the inability to adjust for these varying vaccination statuses on patient level. Since the WHO, apart from the ‘twinning’ quality intervention, also supported hospitals with staff and equipment, it was difficult to judge which factors influenced the observed improvement in quality of care. The available literature clearly highlights the association between the nurse-to-patient ratio in the ICU and mortality [22], and such data would have provided more accurate results. Additionally, other unmeasured and important factors, such as patient comorbidities and severity of illness, might have impacted the association between the twinning intervention and mortality in our study. The severity of illness and comorbidities have been associated with worse outcomes in COVID-19 [23] and should have been adjusted for. However, the reliability of reported comorbidities and disease severity in medical records is generally poor to moderate [24], and in the hospitals taking part in this study, those variables were not systematically and reliably collected in medical records. The lack of reporting of comorbidities is a limiting factor in the assessment of quality of care studies [25]; however, a decision not to request this information was made before this study. Despite the demonstrated general feasibility of the project, there were obstacles to adequate completion of the intervention. While the project aspired to approximately 100–120 visits per pair, roadblocks, lockdowns, fuel crises, and last-minute cancellations considerably reduced the number of performed visits and thus completion of the project. However, the big picture showed the project’s feasibility since most hospitals completed the minimum number of twinning activities and shared their data seamlessly. For full transparency, one hospital was unwilling to share data, possibly due to fear of being stigmatized by other health care facilities or being officially blamed and criticized by other entities. It is also crucial to acknowledge that our project was limited in time. Improvement in quality of care requires constant behavioural management and interventions that are not achieved in short time periods [26]. We theorize that the improvements in quality of care observed could have been greater if the project continued for a longer time, yet further research is needed to provide a definitive answer. As the project is still ongoing across hospitals, further follow-up is needed to evaluate and ensure that the observed effects are long lasting.

### Interpretation within the context of the wider literature

Initiation and implementation of health system studies in LMICs holds multiple challenges and requires continuous learning and change [27]. In fact, the current study is in line with the literature and follows recommendations of pragmatic research paradigms used to address critical health problems [28]. The proposed multi-intervention was able to quantify part of the reality in public sector ICUs and identified and reflected on causes for poor outcomes, further contributing to the growing body of evidence highlighting the adaptability and effectiveness of similar interventions in an LMIC context.

### Implications for policy, practice, and research

Our study demonstrates the feasibility of developing and implementing a monitoring tool in Lebanon, a country with limited evaluation culture. Our approach aligns with the WHO’s call for implementation research, underscoring its relevance and urgency [29]. The challenges found in our project were mainly due to obstacles in providing the number of activities planned for due to an unstable situation in the country. In future studies this must be accounted for, as it highlights the importance of overcoming such barriers, maybe through strategic use of even more local partnerships. Lebanon’s healthcare system exhibits a promising capacity for adaptation [30]. During our study, the staff showed a commendable willingness to adopt and maintain new practices. However, the sustainability of those new practices has not been tested for and future research in similar contexts should focus on the continuity of changes achieved.

## Conclusion

In conclusion, our study showed the feasibility of assessing an intervention aimed at improving quality of care during an ongoing pandemic and despite the absence of a pre-existing data collection system. Our results also showed an association between increased twinning activities and reduced ICU mortality, indicating the potential benefits of interventions aimed at fostering knowledge sharing and capacity building between private and public hospitals.

## Supplementary Material

mzae028_Supp

## Data Availability

The data underlying this article cannot be shared publicly due to restrictions imposed by the individual hospitals that own the data and that participated in the project. These restrictions are in place to protect patient confidentiality and prevent the potential identification of individuals from the data set. However, access to compiled and de-identified data on all hospitals may be granted on a case-by-case basis for researchers who meet the criteria for access to the data.
