# Supplementary material for: Assessing quality of critical care during an ongoing health emergency—a novel approach to evaluate quality of care at Lebanese public ICUs during COVID-19
Source: Int J Qual Health Care. 2024 Apr 6;36(2):mzae028. doi: 10.1093/intqhc/mzae028 (PMC11060481; doi:10.1093/intqhc/mzae028)
Supplement: mzae028_Supp [file mzae028_supp.zip › suppl_data/Language_editing_certificate.pdf]

SPRINGER NATURE
Author Services

Editing Certificate

This document certifies that the manuscript

Assessing quality of critical care during an ongoing health emergency in a resource limited setting - A novel approach to evaluate quality of care at Lebanese public ICUs during COVID-19 pandemic

prepared by the authors

Karim Abou Nader, Ghada Abou Mourad, Georges Chalouhi, Alissar Rady, Johan Von Schreeb, Märit Halmin

was edited for proper English language, grammar, punctuation, spelling, and overall style by one or more of the highly qualified native English speaking editors at SNAS.

This certificate was issued on **November 13, 2023** and may be verified on the SNAS website using the verification code **F166-DC42-71DE-02E4-2D16** .

Neither the research content nor the authors' intentions were altered in any way during the editing process. Documents receiving this certification should be English-ready for publication; however, the author has the ability to accept or reject our suggestions and changes. To verify the final SNAS edited version, please visit our verification page at secure.authorservices.springernature.com/certificate/verify. If you have any questions or concerns about this edited document, please contact SNAS at support@as.springernature.com.

SNAS provides a range of editing, translation, and manuscript services for researchers and publishers around the world. For more information about our company, services, and partner discounts, please visit authorservices.springernature.com.
