# Supplementary material for: Assessing quality of critical care during an ongoing health emergency—a novel approach to evaluate quality of care at Lebanese public ICUs during COVID-19
Source: Int J Qual Health Care. 2024 Apr 6;36(2):mzae028. doi: 10.1093/intqhc/mzae028 (PMC11060481; doi:10.1093/intqhc/mzae028)
Supplement: mzae028_Supp [file mzae028_supp.zip › Supplemental Material.docx]

**Supplemental Material:**

## Supplemental Appendix S1: Quality of Care Checklist

*Missing data gave zero points, and one point was subtracted from the total.*

*The maximum total score was 26 points.*

**Staffing (3 p)**

Responsible doctor a specialist with a critical care diploma 1 p if yes

A2. C4. C5. Nurse/patient ratio 1/2 24/7 1 p if yes on all three

C1. Doctors’ rounds 2 times daily 1 p if yes

**Infrastructure (5 p)**

A10. All patients visible on camera 1 p if yes

A11. Clearly separated clean/dirty areas 1 p if yes

C7. X-ray available 1 p if yes

C8. Echocardiography available 1 p if yes

C9. Time from sampling to result ABG 1 p if < 15 min

**Equipment/Drugs (2 p)**

A12. Intubation equipment readily available 1 p

A13. Intubation drugs readily available 1 p

**Clinical management (14 p)**

A18. Intubated patients with head elevated 1 p if > 80%

A19. Number of prone patients 1 p if one or more

B1. Respiratory rate>12

B2. Minute ventilation >6

B3. Peak pressure>6

B4. Blood pressure >12 1 p if yes on all (B1-4)

B5. Fluid balance in the last 24 hours 1 p if yes

B7. Arterial blood gas daily 1 p if yes

B9. Electrolytes daily

B10. D-dimer at least 2 times/week 1 p if yes on both (B9-10)

B11. Anticoagulation according to guidelines

B12. PPI 1 p if yes on both (B11-12)

B6. Measures after last severely deranged value 1 p if yes

B8. Measures after last pathologic ABG 1 p if yes

A20. Number of unattended alarms during 10 minutes 1 p if no unattended alarm

A16. Any patient with asynchrony with ventilator 1 p if no

A17. Any patient with no peak pressure alarm 1 p if no

C10. System for recording complications 1 p if yes

C14. Regular M&M conferences 1 p if yes

**Protocols (2p)**

A14. Intubation protocol 1 p if yes

A15. Proning protocol 1 p if yes
